# Complications in the first week after stroke: a 10-year comparison

**DOI:** 10.1186/s12883-016-0654-8

**Published:** 2016-08-11

**Authors:** Martina Reiten Bovim, Torunn Askim, Stian Lydersen, Hild Fjærtoft, Bent Indredavik

**Affiliations:** 1 Department of Neuroscience, Faculty of Medicine, NTNU, Norwegian University of Science and Technology, Trondheim, Norway; 2 Regional Centre for Child and Youth Mental Health and Child Welfare, Faculty of Medicine, NTNU, Norwegian University of Science and Technology, Trondheim, Norway; 3 The Norwegian Stroke Register, St. Olavs Hospital, Trondheim, Norway; 4 Stroke unit, St. Olavs Hospital, Trondheim, Norway

**Keywords:** Infarction, Complications, Progressing stroke, Falls, Infection

## Abstract

**Background:**

Complications after stroke have been associated with poor outcome. Modern stroke treatment might reduce the occurrence of complications. The aim of this study was to investigate whether the frequency and type of complications during the first week after stroke has changed in patients treated in a stroke unit in 2013 compared to 2003.

**Methods:**

In total 489 patients in 2003 and 185 patients in 2013 with acute stroke were included and followed prospectively for 1 week, examining the frequency of 12 predefined complications adjusted for severity of stroke. Informed consent was given by all patients or their next of kin.

**Results:**

Mean (SD) age was 77.2 (10.2) and 76.9 (8.5) in 2003 and 2013 respectively, *P* = 0.455. Severity of stroke, measured by the Scandinavian Stroke Scale, was 39.5 (16.8) versus 37.0 (16.4), *P* = 0.011. After adjustment for stroke severity the results showed an odds ratio of 0.64 for experiencing one or more complications in the 2013 cohort versus the 2003 cohort, *P* = 0.035. The subgroup analysis showed that the reduction was only significant in the group with moderate stroke, with 74 % experiencing one or more complications in 2003 compared to 45 % in 2013, *P* < 0.001. Progressing stroke and myocardial infarction occurred significantly less frequent in 2013 than in 2003; the frequency of other complications remained unchanged.

**Conclusions:**

The risk of experiencing one or more complications has decreased from 2003 to 2013. The reduction was most pronounced in patents with moderate stroke with a significant reduction in progressing stroke and myocardial infarction.

## Background

The incidence of stroke seems stable, but the case fatality has decreased in recent decades [1–3]. This trend has been associated with better stroke care (e.g. stroke unit care) [4] and improved preventative treatment of cardiovascular risk factors [1, 3, 5]. Experiencing complications after stroke is, on the other hand, associated with increased mortality and length of hospital stay in acute stroke patients [6]. The most common complications include fever, pain, progressing stroke and infections, but complications such as myocardial infarctions, pulmonary embolisms and cardiac arrest may also occur [6–8]. Most complications have their onset within the first week after stroke [9]. Disability and mortality increase with an increasing number of complications experienced, especially progressing stroke, chest infections and other infections [10, 11].

One important consequence of stroke unit (SU) care has been the prevention and early treatment of complications after stroke, especially those related to immobility [12]. However, studies examining the frequency of complications early after stroke show great discrepancies, with frequency of falls ranging from 2 to 25 % and pneumonia from 9 to 22 % [6, 13]. Such inequalities could be caused by inconsistent definitions of complications, case mix, differences in the completeness of recording, or variation in prevention and treatment of complications among hospitals. Some of these variations might also reflect changes in stroke characteristics and treatment over time. Significant changes in recent decades include the extended use of computer tomography and magnetic resonance imaging, making stroke diagnostics more precise [14], and more frequent use of thrombolysis in acute treatment [15]. Improved physiological homeostasis and early mobilization have also received attention [16].

No systematic studies have thoroughly investigated whether the frequency of complications after stroke have changed in patients treated in dedicated stroke units. The stroke population has also changed in recent decades with a greater proportion of mild strokes probably due to better primary prevention [17]. Because severity of stroke is found to be strongly associated with the risk of complications [9], severity should be considered in comparisons of the frequency of complications in stroke patients over different periods.

The primary aim of this study was to assess the changes in frequency of patients with complications in the acute phase after stroke from 2003 to 2013, adjusted for the severity of stroke. The secondary aims were to assess the changes in complications within the different severity groups and to assess how the different types of complications changed from 2003 to 2013 after adjusting for stroke severity.

Our primary hypothesis was that the frequency of patients with one or more complications has decreased over the last decade in patients treated in a comprehensive SU.

## Methods

### Study design

This prospective observational study assessed the frequency of complications during the first week after stroke, in the SU at Trondheim University Hospital, Norway in 2003 and 2013. The frequency of complications was compared between the 2003- and the 2013-cohort.

### Sample

Patients with acute stroke defined by the World Health Organization (except subarachnoid hemorrhage), treated in the SU in Trondheim and admitted to hospital within 48 h after onset of symptoms (within 24 h for the 2003-cohort) were eligible for inclusion. Patients were fit for inclusion if they were able to understand the Norwegian language and willing to sign informed consent. In keeping with Norwegian consent procedures for patients unable to consent for themselves, such patients were also included if their next of kin did not oppose participation. Patients were excluded if they were transferred to another unit or hospital during the first days or given palliative care (not receiving standard SU treatment), or if they had already been included in the project due to an earlier stroke.

The project was approved by the Regional Committee for Medical and Health Research Ethics in Norway (REC no 2012/1236). The approval included access to data from the 2003-cohort.

### Study setting

All patients were treated in the same comprehensive SU in 2003 and 2013. The SU emphasized a multidisciplinary approach and mobilization to standing or sitting position out of bed within the first 24 h after onset of symptoms. The multidisciplinary team consisted of a physician, a nurse, a physiotherapist, a speech therapist and a member from the early supported discharge team, including an occupational therapist.

Furthermore the treatment consisted of a standardized acute medical treatment program according to the Norwegian Guidelines [18]. Patients were systematically observed and evaluated during the first 72 h, and all received a CT scan within 24 h, preferably within 6 h, after admission. ECG, oxygen saturation and routine blood tests were performed at admission; other diagnostic procedures were performed when indicated. During the first days in the SU, all patients went through a standardized systematic observation and examination of neurologic deficits, blood pressure level, cardiac and pulmonary disorders, temperature, glucose level and fluid and electrolyte balance. The main differences from 2003 to 2013 were a change from intermittent observation 4 to 6 times a day by the nurses on duty in 2003 to continuous monitoring for all patients during the first 24–48 h in 2013. These changes in monitoring procedures were also present during mobilization.

Another difference was the use of thrombolysis in 2003 versus 2013. Thrombolysis was accepted for use in stroke patients in Norway in 2003 [19]. In the initial phase, it was only given to patients included in studies, and within 3 h after stroke. Later, it has been approved for clinical use, and has since 2008 been accepted for use within 4.5 h after stroke.

### Measures

In the 2003 cohort, 14 of the most commonly reported medical complications after stroke were registered [9, 13]. Because the method for measuring troponin T had changed from 2003 to 2013, this complication (elevated Troponin T without MI) was difficult to compare. Pain was also omitted from this study, because the recording of pain was different between the two cohorts, leaving 12 comparable medical complications, listed and defined in Table 1.

**Table 1:** Definition of complications

Progressing stroke	Decrease of more than 2 point on the sum score of the following: consciousness; gaze paresis; arm, hand, or leg strength on the Scandinavian Stroke Scale (SSS) from the first assessment after admission to the assessment 72 h after stroke
Recurrent stroke	New onset of focal or neurologic deficits that cannot be attributed to the presenting lesion and are consistent with World Health Organization definition of stroke
Fever	Temperature ≥38.0 °C at any time during the first week
Seizures	Clinical diagnosis of focal and/or generalized seizure in a previously non-epileptic patient
Falls	
Non-serious falls	Any fall regardless of cause but without serious injury
Serious falls	Falls resulting in fracture or suturing of wounds or prolonged hospitalization
Infections	
Urinary tract infection (UTI)	Clinical symptoms of UTI combined with positive urine dipstick examination for nitrite and/or pyuria
Chest infection	Auscultatory respiratory crackles combined with at least 1 of the following: temperature >38 °C, new purulent sputum, or positive chest radiograph
Acute myocardial infarction (MI)	At least 2 of the following: elevated troponin-T values, chest pain without any other explanation, changes in ECG consistent with MI
Pressure sores	Any skin break or necrosis resulting from pressure of trivial injury (excluding those related to falls)
Thromboembolism	
Deep vein thrombosis	Clinical diagnosis of deep vein thrombosis supported by ultrasound or venography
Pulmonary embolism	Clinical diagnosis supported by computed tomography scan or ventilation/perfusion scan

For each complication, the assessor had to consider whether any of the complications did occur every day during the first week after admission to hospital. An exception was made for body temperature, which was measured only during the first 2 or 3 days and after that only if there was any clinical suspicion of fever and assumed to be normal otherwise. Severity of stroke was assessed by Scandinavian Stroke Scale (SSS) both in 2003 and 2013. The SSS is a reliable scale well validated and simple to perform and therefore used in our study both in 2003 and 2013 [20–22]. The score ranges from 0 to 58 points, where 0 points is the worst possible outcome and 58 is the best and means normal score in all ratings. Functions measured are consciousness, gaze, motor function in affected arm, hand and foot, cognitive function, language, facial palsy, and ability to move. The score has good ability to assess the severity of a stroke and to predict outcome [23, 24].

### Procedure

One trained assessors in 2003 and five trained assessors in 2013 assessed patients arriving at the SU prospectively day-by-day and performed all the registrations. In weekends data was assessed retrospectively by asking the nurses on duty if any complications had occurred, and by assessing the results of the systematic observations of neurological deficits, vital signs and physiological homeostasis in the medical records. As a part of standard SU care, progression of symptoms was assessed 4 to 6 times a day by the nurses on duty and registered in the patient records. To ensure excellent inter-observer agreement in the clinic, all nurses at the ward were very well trained in the scoring system. In the present study, progression was obtained from the patient records and defined as >2 points decrease in sum score from the first assessment after admission until the assessment performed 72 h after stroke. This method of evaluation was similar in 2003 and 2013. Furthermore, meetings were held during the data collection period, to ensure that everyone followed the same procedure. “Day one” of the registration was defined as the day of admission to the SU. Patients hospitalized for less than one week were contacted by telephone at the end of the week and asked if they had experienced any of the relevant complications. Patients who could not be reached by telephone were considered lost to follow-up.

### Statistics

Proportions were analyzed using the Pearson chi squared test. Confidence intervals for difference between proportions were computed using the Newcombe method as recommended [25]. The Mann–Whitney test was used to compare continuous variables. Binary logistic regression was used to compare the frequency of complications adjusted for the severity of stroke by SSS-score, thrombolysis and age as continuous variables. The Cochran-Armitage test for trend, exact mid p version [26] was used to analyze trends between the most common complications and groups of stroke severity. Statistical analyses were performed in SPSS 21, Microsoft Excel 2010 and in StatXact 10.

## Results

In 2003, 664 patients with suspected stroke were admitted to the SU between January 1, 2002 and May 15, 2003. All stroke patients entering the ward during this 16.5 month period were screened for inclusion, and 489 patients were included.

**Fig. 1:**
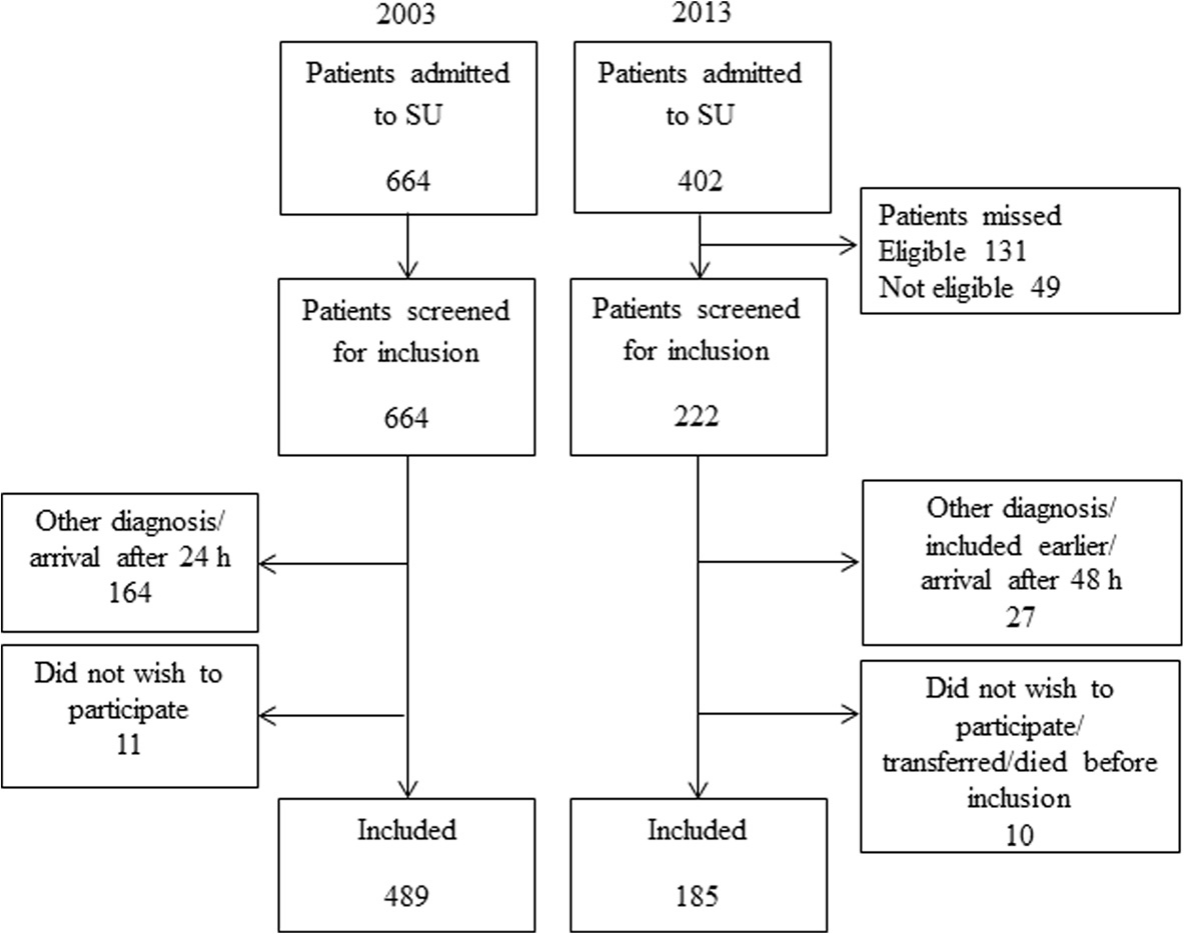
Flow chart for inclusion of patients

In 2013, 402 patients with suspected stroke were admitted to the SU during the inclusion period in 2013, lasting from September 17, 2012 to December 13, 2013, except for a few weeks during the holidays; hence the inclusion period covered 13 months. A final group of 185 patients contributed to the data sample. Of these, 158 (85.4 %) arrived in hospital within 24 h after symptom onset. Ninety-four patients (50.8 %) were discharged during the first week, and were contacted by telephone to complete the registration. Two patients (1.1 %) were not reached by telephone and considered lost to follow-up, and we assumed that no new complications occurred in these patients after discharge. The flow of patients is shown in Fig. 1.

Data from the Norwegian Stroke Register enabled comparison of the 131 patients who were eligible but missed for inclusion with those included in the 2013 cohort. Those missed for inclusion were in mean (SD) aged 79.9 (8.8) years, which was significantly older, *P* = 0.004, with shorter length of stay (LOS) in hospital, mean (SD) 6.3 (6.7) days, *P* < 0.001. Their mean (SD) SSS-score was higher, 39.8 (16.5) points, which was borderline significant, *P* = 0.055. Categorization of SSS-score by severity groups showed significantly more patients in the group of very mild stroke among those missed for inclusion compared with those included, 29.8 % versus 15.1 %, *P* = 0.002.

Table 2 Displays the baseline characteristics of the 2003 and 2013 cohorts, with a significant lower SSS score and mean stay in hospital in the 2013 cohort than in the 2003 cohort. The lower SSS score was caused by a lower proportion with very mild strokes and a higher proportion with moderate strokes in patients included in the 2013 cohort compared with the 2003 cohort. The frequency of prestroke risk factors remained unchanged, except for medical treatment of hypertension which was more common in 2013. Furthermore thrombolytic therapy also increased significantly from 2003 to 2013.

**Table 2:** Characteristics and risk factors of all patients

	2003 cohort (*n* = 489)	2013 cohort (*n* = 185)	*p*-value
Women, *n* (%)^a^	256 (52.4)	88 (47.6)	0.268
Age, mean (SD)^b^	77.2 (10.2)	76.9 (8.51)	0.455
Infarction, *n* (%)^a^	443 (90.6)	161 (87.0)	0.176
Hemorrhage, *n* (%)^a^	46 (9.4)	24 (13.0)	0.176
Scandinavian Stroke Scale, mean (SD)^b^	39.5 (16.8)	37.0 (16.40)	0.011
Days hospital stay, mean (SD)^b^	15 (11.44)	8.5 (8.07)	<0.001
Days hospital stay, median^b^	12	6	
Risk factors, *n* (%)^a^			
Transitory ischemic attack	57 (11.7)	26 (14.1)	0.398
Previous stroke	108 (22.1)	45 (24.3)	0.536
Angina pectoris	86 (17.6)	25 (13.5)	0.200
Myocardial infarction	71 (14.5)	34 (18.4)	0.218
Atrial fibrillation	102 (20.9)	49 (26.5)	0.118
Diabetes	72 (14.7)	28 (15.1)	0.893
Medical treatment for hypertension	190 (38.9)	107 (57.8)	<0.001
Thrombolysis^c^	0	30 (18.6)	<0.001

^a^Pearson chi-squared test was used for comparing dichotomous variables

^b^Mann Whitney U-test was applied on the continuous data

^c^Proportion of patients treated with thrombolysis of all patients with infarction

Table 3 Shows the frequencies of the most common complications in each stroke severity group. The tests for trend showed that risk of each of these complications, with a possible exception for falls, increased with increasing stroke severity. Recurrent stroke, seizures, pressure sores and thromboembolisms were experienced by less than 4 % of the patients in both cohorts.

**Table 3:** Frequency of and trend for the most common complications in the 2003 and 2013 cohort

		2003							2013					
Stroke severity	*n*	Fever	Chest infection	Prog. stroke	MI	UTI	Falls	*n*	Fever	Chest infection	Prog. stroke	MI	UTI	Falls
		*n* (%)	*n* (%)	*n* (%)	*n* (%)	*n* (%)	*n* (%)		*n* (%)	*n* (%)	*n* (%)	*n* (%)	*n* (%)	*n* (%)
Very severe	61	34 (55.7)	29 (47.5)	18 (29.5)	8 (13.1)	12 (19.7)	2 (3.3)	26	18 (69.2)	11 (42.3)	0	1 (3.8)	5 (19.2)	3 (11.5)
Severe	58	32 (55.2)	14 (24.1)	12 (20.7)	8 (13.8)	9 (15.5)	4 (6.9)	24	11 (45.8)	6 (25.0)	3 (12.5)	0	4 (16.7)	5 (20.8)
Moderate	111	23 (20.7)	4 (3.6)	41 (36.9)	4 (3.6)	31 (27.9)	20 (18.0)	58	10 (17.2)	1 (1.7)	1 (1.7)	0	8 (13.8)	10 (17.2)
Mild	124	19 (15.3)	7 (5.6)	13 (10.5)	1 (0.8)	20 (16.1)	10 (8.1)	49	5 (10.2)	0	3 (6.1)	0	5 (10.2)	3 (6.1)
Very mild	135	8 (5.9)	1 (0.7)	6 (4.4)	1 (0.7)	6 (4.4)	4 (3.0)	28	2 (7.1)	0	2 (7.1)	0	0	1 (3.6)
Total number	489	116 (23.7)	55 (11.2)	90 (18.4)	22 (4.5)	78 (16.0)	40 (8.2)	185	46 (24.9)	18 (9.7)	9 (4.9)	1 (0.5)	22 (11.9)	22 (11.9)
*p*-value for trend^a^		<0.001	<0.001	<0.001	<0.001	0.001	0.34		<0.001	<0.001	0.46	0.070	0.025	0.091

Very severe: SSS = 0–14, severe: SSS = 15–29, moderate: SSS = 30–44, mild: SSS = 54–51, very mild: SSS = 52–59

^a^Cochran-Armitage test for trend

Table 4 Shows the risk of complications in each stroke severity group during the first week after stroke onset in the 2003 and 2013 cohort. In all the stroke categories very mild to severe, this risk was lower in 2013 compared to 2003. This reduction was statistically significant (*P* < 0.001), and most pronounced for moderate strokes, where 73.9 and 44.8 % of the patients in 2003 and 2013, respectively, experienced one or more complications.

**Table 4:** Frequency of and risk difference for developing complications according to stroke severity on admission

	2003			2013				
Stroke severity	No. of patients	One or more complications		No. of patients	One or more complications		RD	95 % CI
		*n*	%		*n*	%		
Very severe	61	50	82.0	26	24	92.3	−0.10	−0.23 to 0.08
Severe	58	45	77.6	24	16	66.7	0.11	−0.09 to 0.33
Moderate	111	82	73.9	58	26	44.8	0.29	0.43 to 0.14
Mild	124	50	40.7	49	13	26.5	0.14	−0.02 to 0.27
Very mild	135	25	18.4	28	5	17.9	0.01	−0.18 to 0.13
*p*-value for trend^a^			<0.001			<0.001		

Very severe: Scandinavian Stroke Scale (SSS) = 0–14, severe: SSS = 15–29, moderate: SSS = 30–44, mild: SSS = 54–51, very mild: SSS = 52–59

*RD* risk difference

^a^Cochran-Armitage test for trend

Table 5 Shows the odds ratio for developing the most frequent complications in 2013 compared to 2003 cohort, adjusted for age, thrombolysis and stroke severity. Both progressing stroke and myocardial infarction (MI) seem to occur less frequently in the 2013 cohort than the 2003 cohort. In the 2013 cohort, MI was reported for one patient only, and this patient had received thrombolysis. Hence, we did not adjust for thrombolysis in this case. The frequency has not changed significantly for any of the other complications, and falls might even tend to be more common in the 2013 cohort. The odds ratio for risk of complications in 2013 compared with 2003, adjusted for age, thrombolysis and stroke severity, was 0.64, *P* = 0.035.

**Table 5:** OR for development of complications in the 2013 cohort compared with the 2003 cohort

Complication	OR	95 % CI for OR	*p*-value
Fever	0.94	0.59 to 1.52	0.81
Chest infection	0.51	0.24 to 1.09	0.08
Progressing stroke	0.26	0.13 to 0.53	<0.001
MI^a^	0.09	0.01 to 0.70	0.02
UTI	0.64	0.36 to 1.13	0.12
Falls	1.56	0.88 to 2.79	0.13
One or more complications^b^	0.64	0.43 to 0.97	0.035

*OR* odds ratio, *SSS* Scandinavian stroke scale, *MI* myocardial infarction, *UTI* urinary tract infection

^a^Not adjusted for thrombolysis

^b^Represents all 12 complications

## Discussion

To our knowledge, this is the first study to show 10-year time trends for complications in patients treated in a dedicated comprehensive stroke unit. The study shows that complications are still common in the acute phase after stroke, but are generally less common in 2013 than in 2003, also after adjusting for differences in stroke severity between the two cohorts. The reduction in complications was most pronounced in those with moderate stroke. Regarding specific complications, the reduction was significant for progressing stroke and myocardial infarction. There was also a trend towards fewer chest infections.

Despite an overall reduction in the risk of experiencing one or more complications from 2003 until 2013, it seems that prevention of complications was not improved for patients with severe strokes or mild strokes. However, patients with moderate strokes showed a 29 % risk reduction over this period. One possible explanation is the improved monitoring and stronger focus on physiological homeostasis that has been implemented in the ward over the past years, and an increased use of thrombolysis. In the present study, the effect of thrombolytic therapy on complication risk was assessed by adjusting for thrombolysis in the regression analysis.

The significant decrease in progressing stroke particularly in patients with moderate and severe stroke indicates that early detection of risk and treatment of progression might have improved considerably. It is a strength in our study, that progression was defined and assessed in the same way by well-trained nurses on duty both in 2003 and in 2013. The inter-observer agreement has shown to be quite good using this method and the reduction observed reflects probably a real difference [20]. Patients with moderate stroke seem to have the most pronounced reduction of frequency of progressing stroke. The reason for less reduction in the severe group might be that progression of symptoms in this group quite often is due to brain edema, which might not be reduced by monitoring and better control of the physiological homeostasis. Mild strokes on the other hand have quite a low risk of progression and due to low number of events it will be difficult to find differences in this group. Hence, the patients with moderate stroke might be the group where system factors like blood pressure, oxygen saturation and temperature are likely to be of most importance for progression. In a review from the Cochrane Library they also found a trend towards reduced rate of neurological complications like progressing stroke in stroke units with continuous monitoring which is quite similar to our result [27]. However in the Cochrane review no stratification by severity of the stroke was performed.

Previous studies have found frequencies of progressing stroke ranging from 11 to 43 % [28, 29]. The great variability in the reported incidence has different reasons. One important reason is that it has been difficult to construct precise definitions of exacerbations of a stroke and various terms such as “progressive stroke”, “early neurologic deterioration” or “stroke in evolution” have been used and have not made studies of these condition easier [30]. Furthermore the use of different scales, cut-off values and time perspectives in the definitions makes conclusions and comparisons even more difficult [31]. Hence, a consensus on the definition of progressing stroke is needed to enhance further research within this field.

A significant decrease was also seen for acute MI, from 22 cases (4.5 %) in 2003 to only one case (0.5 %) in 2013. This very low incidence makes it difficult to conclude on the association between MI and stroke severity and to adjust for thrombolysis, as the single patient receiving MI in 2013 received thrombolysis while none of the patients in 2003 did. The method for measuring Troponin T was changed between 2003 and 2013, but the criteria for Troponin levels according to MI were quite similar. Hence the reduction of MI is probably also a real difference.

The significant decrease in the occurrence of MI is supported by an Austrian study [32] carried out from 2006 to 2013, where MI was found in 1 % of stroke patients during hospital stay. Maintenance of physiologic homeostasis is one important factor contributing to the good outcome of SU care [16], and improvement in such regulation might influence the risk of post-stroke MI. Another important factor is continuous monitoring of patients (ECG and oxygen saturation) which makes it easier to detect signs of ischemia on the heart (atrial fibrillation and heart failure leading to hypoxia) [4], so that prophylactic treatment can be initiated early in risk patients. A report from the Norwegian Institute of Public Health shows that the incidence of first-time-MI is decreasing in the general elderly Norwegian population, which possibly also has influenced our findings [33].

In the 2003 cohort fever, chest infections, progression of stroke, MI and UTI occurred significantly more often in patients with severe than with milder strokes but in the 2013 cohort such an association to severity occurred only for fever, chest infection and UTI and a trend for MI while progressing stroke had no such association to severity. For MI with only one case, association to severity is not possible to assess. It is not possible to conclude whether the reduction in frequency of progression of symptoms have contributed to less association to severity of the stroke.

The use of thrombolysis in 30 patients (16 %) in 2013 versus no patients (0 %) in 2003 might have influenced the complication rate. However, after adjusting for thrombolytic therapy no significant changes were found. Hence, thrombolytic therapy has probably not contributed very much to the changes in complication rate between 2003 and 2013.

A major strength of this study was that patients were followed up daily through a prospective design, minimizing the probability of omitting incidents of complications. Having a comparable cohort admitted to hospital 10 years ago reinforces the comparison, as the definitions of complications were the same. The studies were performed in the same SU which offers evidence-based treatment according to the Norwegian guidelines.

The study had some limitations. Fewer patients were screened for inclusion in 2013 compared to 2003. This was due to a reduction in the entrance area and also to a reduction in the number of beds for the SU at St. Olavs Hospital between 2003 and 2013. Furthermore, a considerable amount of patients were missed for inclusion. LOS was significantly shorter in the 2013 cohort, but those who were missed for inclusion in 2013 had an even shorter LOS. Some patients with mild stroke were discharged so rapidly that it was difficult to get time to include them in the study. This probably explain why there were significantly fewer patients with very mild stroke in those included in 2013 than in those who were missed. Hence, we cannot say that the differences in stroke severity between our 2003 and 2013 cohort represent an actual difference in these populations. This is taken into consideration by adjusting for stroke severity in all analyses, as stroke severity strongly influences the risk of experiencing complications in the acute phase. Hence, we do not think the differences in stroke severity influence the results very much. One should consider if the shortened LOS and the follow-up by telephone in 2013 have limited the ability to capture some complications that might occur after discharge. However, most complications occur within the first days after stroke [9], and the follow-up via telephone was performed thoroughly by describing and asking for each complication specifically, hence we do not believe that these features have made a huge impact on our results.

Another limitation was that the time limit for arrival in hospital after onset of symptoms was expanded from 24 h in 2003 to 48 h in 2013, to include sufficient patients in the study and increase the power. This increases the risk of missing complications that occurs after the stroke but before admission to hospital, as these were not registered. This matter especially for progressing stroke and other temporary complications such as falls and seizures; as most of the other complications would still be present by admission and therefore be registered. However 85 % were admitted within 24 h and a post hoc analysis showed almost identical results when patients arriving after 24 h were excluded from the analyses, and this is considered not to have affected our results significantly.

## Conclusion

Complications are still common in the acute phase after stroke in patients treated in a comprehensive stroke unit. However, after adjusting for age, thrombolysis and stroke severity, the odds of experiencing one or more complications have decreased significantly from 2003 to 2013. The reduction was greatest for progressing stroke and myocardial infarction. Overall reduction in complications was mainly seen for patients with moderate stroke. The reduction in complication rate cannot be explained by the introduction of thrombolytic therapy but might be explained by an increased focus on continuous monitoring and stabilizing physiological homeostasis over the past 10 years.

## Abbreviations

CT, computed tomography; ECG, electrocardiogram; LOS, length of stay; MI, myocardial infarction; RD, risk difference; SSS, scandinavian stroke scale; SU, stroke unit; UTI, urinary tract infection
